# Exploring the benefits of altered states of consciousness and reduced experiential avoidance for mental well-being: insights from a global study on high ventilation breathwork

**DOI:** 10.3389/fpsyg.2026.1777341

**Published:** 2026-07-30

**Authors:** Lena Erdmann, Barbara Breitenstein, Maria Badanova, Lennard Telschow, Martha Havenith, Isabel Dziobek

**Affiliations:** 1 Department of Sports and Health Sciences, University of Potsdam, Potsdam, Germany; 2 Department of Psychology, Humboldt-Universität zu Berlin, Berlin, Germany; 3 German Center for Mental Health (DZPG), Berlin, Germany; 4 Max Planck School of Cognition, Max Planck Institute for Human Cognitive and Brain Sciences, Leipzig, Germany; 5 Department of Psychiatry and Psychotherapy, Charité - Universitätsmedizin Berlin, corporate member of Freie Universität Berlin and Humboldt-Universität zu Berlin, Berlin, Germany; 6 Ernst Strüngmann Institute, Frankfurt, Germany

**Keywords:** altered states of consciousness, breathwork, challenging experiences, emotional breakthrough, experiential avoidance, high ventilation, hyperventilation, well-being

## Abstract

**Background:**

Research suggests that high ventilation breathwork (HVB) improves mental health by reducing experiential avoidance (EA) through altered states of consciousness (ASCs). The present study examined whether EA during participants’ most recent breathwork session predicted mental well-being afterward. Additionally, the study examined the relationships between EA, ASCs, emotional breakthroughs, and mentally challenging experiences (e.g., negative emotions or thoughts) during the session and mental well-being afterward.

**Methods:**

This cross-sectional, international online study was conducted in German and English and included a final sample of *N* = 324 participants from 23 countries of residence. Participants reported on their most recent breathwork experience and completed validated questionnaires, including the APEQ-S, 11D-ASCs, EBI, and WEMWBS, as well as a self-developed challenging experiences scale. Multiple linear regressions and mediation analyses were conducted.

**Results:**

During the session, ASCs significantly predicted lower EA. In turn, lower EA during the session significantly predicted higher mental well-being after the session. Moreover, higher ASC ratings and lower ratings for challenging experiences significantly predicted higher mental well-being after the session. EA did not significantly predict emotional breakthroughs, nor did it mediate the relationships between ASCs or challenging experiences and mental well-being.

**Conclusion:**

Overall, the findings suggest that reduced EA and more intense ASCs during HVB enhance mental well-being. Blissful states within ASCs predicted lower EA, highlighting their potential in fostering openness and engagement during HVB.

## Introduction

High ventilation breathwork (HVB) has attracted growing interest as a therapeutic tool in the context of the psychedelic renaissance and the expanding scientific research on mind-and-body practices (Hendy, 2021; Corso et al., 2023; Fincham et al., 2023b; Fincham et al., 2023a). In the present study, HVB is defined as practices “that involve manipulating the manner in which one breathes” (Oxford English Dictionary, 2023) by increasing the rate and/or depth of ventilation (Fincham et al., 2023b). The most prominent types of HVB include Holotropic Breathwork (Grof® Breathwork), Holorenic Breathwork, Conscious Connected Breathwork, and Rebirthing Breathwork (Fincham et al., 2023a).

Many studies on HVB have reported improvements in mental health-related constructs, such as mental well-being, life satisfaction, sense of meaning in life, mindfulness-related constructs, symptoms of depression, anxiety and post-traumatic stress disorder (PTSD), and stress-related symptoms (Blake, 2026; Puente, 2014b; Cervantes and Puente, 2014; Miller and Nielsen, 2015; Uthaug et al., 2022; Khubsing et al., 2024; Havenith et al., 2025; De Wit and Cruz, 2021). However, randomised controlled trials (RCTs) are scarce, and findings across different studies are not entirely consistent, with some studies failing to demonstrate significant mental health benefits (Banushi et al., 2023; Fincham et al., 2024). Additionally, the mechanisms underlying HVB’s effects remain underexplored (Fincham et al., 2023a). Therefore, the present study investigates potential mechanisms of HVB’s mental health benefits, focusing on experiential avoidance (EA) and Altered States of Consciousness (ASCs).

Several theories have been proposed to explain how HVB impacts mental health. As HVB is often conducted in groups and led by facilitators, positive social interactions may enhance mental health outcomes (Sun et al., 2020; Nakamura et al., 2021; Nakamura and Iwakabe, 2017). Physiologically, hyperventilation induces short-term sympathetic activation but may reduce stress and inflammation in the long term, resembling the health-promoting effects of physical exercise (Zwaag et al., 2022; Kox et al., 2014; Kopplin and Rosenthal, 2022). Moreover, sustained hyperventilation reduces carbon dioxide levels, leading to respiratory alkalosis (Watts et al., 2018; Fincham et al., 2023a). This shift alters central nervous system functions by increasing neuronal excitability and reducing inhibitory GABAergic transmission (Li et al., 2011; Watts et al., 2018). If enhanced ventilation is continued for at least 8–15 min, these metabolic changes can affect cognitive and perceptual processes, often leading to ASCs (Agadzhanyan et al., 2003; Fincham et al., 2023a).

ASCs can manifest themselves in a wide range of acute effects on perception, emotion, and cognition. They may result in feelings of oceanic boundlessness, ego dissolution, universal interconnectedness, and mystical experiences (Ko et al., 2022). These phenomena during HVB can bear similarities to ASCs induced by the ingestion of psychedelic substances such as lysergic acid diethylamide (LSD), psilocybin, or other hallucinogens (Eyerman, 2013; Puente, 2014a; Jacob, 2020; Bahi et al., 2023; Khubsing et al., 2024; Havenith et al., 2025). Notably, mystical experiences or ego dissolution during psychedelic-assisted therapy are significant correlates or predictors of improved mental health outcomes, including depression, substance use disorder, and cancer-related distress (Ko et al., 2022; Van Amsterdam and Van den Brink, 2022).

Although there is strong evidence for the beneficial effects of ASCs in appropriate contexts (Carhart-Harris et al., 2018; Levin et al., 2024; Cavarra et al., 2022), it remains unclear how exactly ASCs lead to improved mental health outcomes. One theory is that the downregulation of the default mode network allows for psychological flexibility (Gattuso et al., 2022; Cho et al., 2025). Moreover, ASCs during HVB can aid the processing of mentally challenging experiences (i.e., difficult emotions, thoughts, or memories) by eliciting emotional breakthroughs marked by cathartic emotional release, insight, or self-acceptance (Roseman et al., 2019; Aday et al., 2020; Grof and Grof, 2010; Uthaug et al., 2022). However, one of the most prominent theories is that ASCs during HVB may foster healthier engagement with such experiences by reducing EA, as ASCs may reduce EA and EA may lessen the likelihood of mentally challenging experiences to exacerbate (Wolff et al., 2020; Carhart-Harris et al., 2021; Mertens et al., 2020; Belser et al., 2017; Gasser et al., 2014; Watts et al., 2017; Banushi et al., 2023; Davis et al., 2020; Rhinewine and Williams, 2007).

According to the founder of Acceptance and Commitment Therapy (ACT; Hayes et al., 1996; Hayes et al., 1999), EA consists of attempts to avoid challenging mental experiences and can persist even when it is harmful, interferes with personal values, and intensifies suffering. Avoiding stimuli that signal danger is often essential for survival (Arnaudova et al., 2017), whether it involves keeping a safe distance from aggressive wild animals, seeking shelter during thunderstorms, or wearing a seatbelt while driving. However, EA can become maladaptive when individuals habitually avoid relatively safe or long-term beneficial stimuli (Arnaudova et al., 2017). Opportunities for the modification of dysfunctional cognitions, such as fearful beliefs, are limited when direct experiences are avoided (Cookson et al., 2019). Moreover, short-term relief of discomfort is achieved through the avoidance of unpleasant stimuli, thereby increasing the likelihood that the avoidance behaviour will persist while restricting people’s activities, leading to diminished mental health (Kashdan et al., 2006; Shari et al., 2019).

In the context of psychedelic-assisted therapy, Wolff et al. (2020) have proposed a cognitive-behavioural model which suggests that the therapeutic effects following ASCs are associated with shifts from excessive EA towards greater acceptance, especially when mentally challenging experiences occur. According to their model, EA within psychedelic experiences is reduced through three main mechanisms: (1) the relaxation of avoidance-related beliefs (such as “anxiety is dangerous”) due to enhanced neural plasticity, (2) motivational processes (i.e., operant conditioning in which avoidance leads to enhanced aversive experiences and acceptance leads to relief), and (3) avoidance free exposure, which is possible due to the combination of the first two processes and the elicitation and intensification of emotional experiences. Wolff et al. (2020) suggest that ASCs could reduce EA and that EA might mediate the relationship between mentally challenging experiences and improved mental health outcomes following psychedelic experiences.

A recent double-blind RCT among individuals with depression supports this theory by showing that improvements in mental health, such as mental well-being, symptoms of depression, anxiety, and suicidal ideation, occurred through reductions in EA following psilocybin therapy, but not within the escitalopram treatment group (Zeifman et al., 2023). Notably, these reductions in EA were found to be predicted by acute experiences of ego dissolution and psychological insight during the treatment (Zeifman et al., 2023). Building on this research, the present study explores the relationships among ASCs, mentally challenging experiences, and EA during HVB, as well as mental well-being afterward. Avoiding mentally challenging experiences during ASCs in HVB may hinder a possible long-term resolution and integration of these challenges, leading to diminished subsequent mental well-being, as seen in psychedelic-assisted therapy (Wolff et al., 2020; Zeifman et al., 2023).

Although theoretical accounts and emerging empirical evidence suggest that EA may hinder psychological outcomes in psychedelic-assisted therapy (Wolff et al., 2020; Wolff et al., 2022; Zeifman et al., 2023), its role in HVB has only been proposed and remains largely unexamined (Banushi et al., 2023; Rhinewine and Williams, 2007). Accordingly, the present study explored how HVB may promote mental well-being by focusing on the dynamic interplay among EA, ASCs, mentally challenging experiences, and emotional breakthrough experiences during the session. Specifically, we investigated whether more intense ASCs were associated with lower EA levels during HVB and, in turn, with greater post-session mental well-being. In addition, we tested whether EA mediated the relationships between ASCs or mentally challenging experiences and mental well-being in the context of HVB. Finally, we proposed that low EA may be a key precondition for emotional breakthrough experiences, as EA may inhibit the insights and acceptance central to such experiences (adapted from Wolff et al., 2020; Wolff et al., 2022).

The corresponding hypotheses are as follows: *H1*: Higher levels of EA during the session predict lower levels of mental well-being after the session (primary hypothesis); *H2*: Higher levels of ASCs predict lower levels of EA during the breathwork session; *H3*: EA mediates the relationship between ASCs and mental well-being; *H4*: EA mediates the relationship between mentally challenging experiences during the session and mental well-being afterwards; *H5*: Lower levels of EA predict higher levels of emotional breakthrough experiences during the session.

## Methods

### Participants

*A priori* power analysis conducted with G*Power 3.1 (Faul et al., 2009) indicated a required sample size of at least 152 participants to detect a small to medium effect in multiple linear regressions (*f*^2^ = 0.085; *α* = 0.05; 1–*β* = 0.90). Recruitment of participants was conducted from March to June 2024 through various channels, including university recruitment, email newsletters, social media announcements, flyers, posters, and word-of-mouth. Calls for participation were disseminated via email and the recruitment system of Humboldt-Universität zu Berlin. The final sample consisted of *N* = 324 participants.

Participants were eligible if they were at least 18 years old, proficient in German or English, had attended at least one HVB session within the past five years, and reported a continuous accelerated breathing phase of ≥15 min, as the manifestation of ASCs is likely to be more pronounced when enhanced ventilation is practiced for at least 8–15 min (Agadzhanyan et al., 2003; Fincham et al., 2023a). Participants were excluded if any of the following criteria were met: (1) unusually fast response behaviour, operationalised as a TIME_RSI score ≥ 2 in SoSci Survey (Leiner, 2024), (2) disclosing the use of a psychedelic substance during the most recent breathwork session, (3) responding to the feedback request with “please discard my responses,” and/or (4) indicating poor memory of the reported experience.

### Procedure and ethical considerations

This cross-sectional international online survey aimed to gather insights from participants about their most recent HVB session in a naturalistic setting. Implementation was facilitated in German and English on the SoSci Survey platform (Leiner, 2024). The average time to complete the survey was 19.50 min (*SD* = 7.67). The study was preregistered at the Center for Open Science (Erdmann et al., 2024).

The study was approved by the Ethics Committee at Humboldt-Universität zu Berlin and was conducted in accordance with the principles of the Declaration of Helsinki (World Medical Association, 2013). The study was conducted anonymously, and participants were asked to provide informed consent before participation. After completing the survey, participants had the opportunity to enter a lottery for a chance to win one of ten €50 prizes, which were randomly awarded to all lottery entrants.

### Measures

#### Experiential avoidance

To assess perceived EA during breathwork, 7 items of the short version of the Acceptance/Avoidance-Promoting Experiences Questionnaire (APEQ-S) were included (Wolff et al., 2022). The APEQ-S is an instrument designed to measure aspects of psychedelic experiences that are associated with changes in psychological flexibility on a scale from 0% (“No, not at all”) to 100% (“Yes, extremely or absolutely”). The questionnaire includes the scales “Acceptance-Related Experience” and “Avoidance-Related Experience,” which have been shown to be two overall independent constructs (Wolff et al., 2022). In the current study, only the subscales “Pro-Avoidance Insights” (3 items) and “Avoidant Responses” (4 items) of the scale “Avoidance-Related Experience” were included. The subscales “Pro-Avoidance Insights” (*α_cron_* = 0.70 in the English, and *α_cron_* = 0.78 in the German sample) and “Avoidant Responses” (*α_cron_* = 0.78 in the English, and *α_cron_* = 0.84 in the German sample) have demonstrated acceptable to high internal consistency (Wolff et al., 2022).

#### Mental well-being

Perceived change in mental well-being was measured using the Warwick-Edinburgh Mental Well-Being Scale (WEMWBS) by Tennant et al. (2007), which is designed to capture the affective and functional aspects of mental well-being. The scale consists of 14 positively worded items, rated on a Likert scale from 1 to 5. In relation to Joseph et al. (2012), the answer options were revised to capture retrospectively perceived changes in participants’ mental well-being after HVB, ranging from 1 (“much less so than before”; previously: “none of the time”) to 5 (“much more so than before”; previously: “all of the time”). Accordingly, the instructions were changed to “Please […] rate if you noticed a change in yourself in the following days (up to 2 weeks) after doing the breathwork session.” The WEMWBS shows excellent internal consistency (*α_cron_* = 0.91), high test–retest reliability (*r* = 0.83), and good construct validity (Tennant et al., 2007). The adjusted WEMWBS questionnaire showed excellent internal consistency in the present study (German: *α_cron_* = 0.92; *n* = 240; English: *α_cron_* = 0.93, *n* = 83). In addition to the WEMWBS, we included a single item assessing perceived lasting change in mental well-being: “Do you believe that this breathwork session has led to a lasting change in your sense of well-being?” which was rated from 0% (decreased very much) to 100% (increased very much).

#### Altered states of consciousness

The intensity of subjectively experienced ASCs during HVB was assessed using the 11-Dimensional Altered States of Consciousness Rating Scale (11D-ASCs, Studerus et al., 2010). The 11D-ASCs is a scale of 42 items evaluating changes in an individual’s consciousness that are commonly experienced during activities such as psychedelic use from 0 (“no more than usual”) to 100 (“much more than usual”). In the interest of brevity, only the positive dimensions “experience of unity” (5 items); “spiritual experience” (3 items); “blissful state” (3 items) and “insightfulness” (3 items) were included. Acceptable to excellent internal consistency can be observed across each subscale (from *α_cron_* = 0.73 to *α_cron_* = 0.91; Studerus et al., 2010). In the German sample of the present study, the subscales demonstrated acceptable to high internal consistency (*α_cron_* = 0.78–0.89, n = 240), whereas the English sample demonstrated high to excellent internal consistency (αcron = 0.84–0.91, *n* = 84).

#### Mentally challenging experiences

Regarding the intensity of mentally challenging experiences during the session, the survey incorporated four self-developed items: (1) “Feeling down, sad, or hopeless,” (2) “Feeling nervous, anxious, or on edge,” (3) “Feeling exposed, unprotected, or fragile,” (4) “Experience of flashbacks or re-experiencing traumatic events.” These items were formulated following qualitative interviews with HVB facilitators and individuals who were new to breathwork and are referred to as the Challenging Experiences Scale in the present study (CES). Participants were asked to rate the intensity on a scale ranging from 0% (“Not at all”) to 100% (“Severe”), in increments of 10%. The self-designed scale showed acceptable internal consistency in the present study (German sample: *α_cron_* = 0.78, *n* = 240; English sample: *α_cron_* = 0.72, *n* = 84).

#### Emotional breakthrough

To capture emotional breakthrough during HVB, the Emotional Breakthrough Inventory (EBI) was used: A 6-item scale designed to measure emotional release/breakthrough during an acute psychedelic experience (Roseman et al., 2019). Subjects rated the extent to which each statement was experienced during the intervention from 0 (“Not at all”) to 100 (“Yes, very much so”). The EBI shows excellent internal consistency (*α_cron_* = 0.93; Roseman et al., 2019). In the absence of a validated German EBI, the measure was translated into German and subsequently back-translated into English to verify consistency between versions. The German EBI showed excellent internal consistency in the present study sample (*α_cron_* = 0.92, *n* = 238).

### Statistical analyses

Analyses were conducted using SPSS 28.0 (IBM Corp, 2020). Multiple linear regressions were used to test *H1*, *H2*, and *H5*, with bias-corrected bootstrapping (3,000 resamples) to derive confidence intervals and *p*-values (Freedman and Peters, 1984). Separate sensitivity analyses were conducted for the main findings by additionally adjusting the primary models for (1) memory clarity and time since session and (2) BW type using dummy-coded variables. Mediation analyses (*H3*, *H4*) were performed using PROCESS v4 (Hayes, 2018), applying ordinary least squares regression and bootstrapping with 5,000 samples and heteroscedasticity-consistent standard errors. Effects were considered significant when the confidence interval did not include zero. Assumptions of linearity, homoscedasticity, normality, and multicollinearity were evaluated using standard diagnostics (scatterplots, residual plots, Shapiro–Wilk test, tolerance, VIF). Independence of residuals was assessed via the Durbin–Watson statistic. Outliers in residuals (> 3 *SD*) or cases with Cook’s distance > 1 were excluded. Effect sizes for regressions were interpreted following Muller (1989): |*R*^2^| = 0.02: small/weak, |*R*^2^| = 0.13: moderate/medium, |*R*^2^| = 0.26: strong/big explanation of variance. A significance level of *α* = 0.05 was applied to all tests.

## Results

### Demographics and breathwork session characteristics

In total, the survey was accessed 589 times. 387 volunteers provided informed consent, of whom 346 completed the relevant measures. Twenty-two participants were excluded due to self-requested data deletion (*n* = 7), unclear recall of the reported session (*n* = 7), or being under the influence of a psychoactive substance during the session (*n* = 8). Missing data were minimal (*M* = 0.12%, *SD* = 0.08%, range: 0–0.31% per scale); therefore, cases with missing values were listwise excluded from analyses involving the respective scale. Of the included participants, 74.1% completed the German and 25.9% the English version of the survey. The participants had a mean age of 39 years, with an age range of 18 to 77 years. The sample included 214 participants identifying as female (66.0%), 97 identifying as male (29.9%), and 13 identifying as diverse or other (4.0%). Participants resided in 23 countries, with 82% living in predominantly German-speaking regions (Germany, Switzerland, Austria). Educational attainment in the sample was high, with 82.1% of participants holding at least a bachelor’s degree. Additional demographic characteristics are presented in Table 1.

**Table 1:** Demographics and characteristics of included participants.

Participant characteristics (*N* = 324)		*M* (*SD*)	Range
	Age in years	38.98 (11.73)	18–77
	Number of prior breathwork sessions*	20.84 (49.82)	0–600
	Number of lifetime mental health diagnoses	1.26 (0.73)	0–6

Participant characteristics (*N* = 324)		*n*	Percentages
Gender	Female	214	66.0%
	Male	97	29.9%
	Diverse	12	3.7%
	Other	1	0.3%
Country	Germany	247	76.2%
	Austria	10	3.1%
	Switzerland	8	2.5%
	Other**	59	18.2%
Educational level	Secondary education (9–10 school years)	13	4.0%
	High school diploma/Abitur	45	13.9%
	Bachelor	61	18.8%
	Master	134	41.4%
	PhD/Doctoral	32	9.9%
	Other	39	12.0%
Lifetime diagnosis of mental disorder	Any diagnosis	109	33.6%
	Depression	73	22.5%
	Post-traumatic stress disorder	27	8.3%
	Anxiety disorder	24	7.4%
	Neurodevelopmental disorder	15	4.6%
	Eating disorder	12	3.7%
	Somatoform disorder	11	3.4%
	Bipolar disorder	3	0.9%
	Schizophrenia/Psychotic disorder	3	0.9%
	Other	15	4.6%

*n = 321 participants answered this item; median = 6 prior breathwork sessions. **Other countries: Australia, Belgium, Canada, Denmark, Egypt, Finland, France, Hungary, Ireland, Italy, Luxembourg, México, Netherlands, Northern Cyprus, Norway, Portugal, Romania, Spain, United Kingdom, United States of America (Range 0.3–1.9%).

More than half of the participants reported on a breathwork session that had taken place within the past 3 months, and 74% recalled the session very clearly to completely clearly. Together, Conscious Connected Breathwork and Holotropic Breathwork® accounted for roughly 60% of participants’ sessions, making them the most frequently practiced breathwork schools. On average, sessions involved two facilitators (*SD* = 1.81) and 17 other participants (*SD* = 35.23). About 75% indicated that the active breathing phase lasted between 15 and 60 min (see Table 2 for details).

**Table 2:** Characteristics of participants’ most recent breathwork session.

Session characteristics (*N* = 324)		*n*	Percentage
Reported time interval since the session	0–3 months	167	51.5%
	4–6 months	60	18.5%
	7–12 months	37	11.4%
	13–24 months	28	8.6%
	Between 2 and 3 years ago	14	4.3%
	Between 3 and 4 years ago	12	3.7%
	Between 4 and 5 years ago	6	1.9%
Rated clarity of memory of the session	Completely clear	115	35.5%
	Very clear	125	38.6%
	Clear	48	14.8%
	Somewhat clear	36	11.1%
Type of breathwork	Conscious Connected Breathwork	99	30.6%
	Holotropic Breathwork®	92	28.4%
	Circular Breathing	23	7.1%
	Shamanic Breathing	11	3.4%
	The Wim Hof Method	6	1.9%
	Psychedelic Breath	5	1.5%
	Rebirthing Breathwork	4	1.2%
	Transformational Breathwork	4	1.2%
	Other:	48	14.8%
	I do not know	33	10.2%
Duration	15–30 min	101	31.2%
	30–60 min	141	43.5%
	60–120 min	54	16.7%
	> 120 min	28	8.6%
Online or in person	Online	51	15.7%
	In-person	273	84.3%
Presence of music	Music	288	88.9%
	No music	36	11.1%
Occurrence of touch	Touch by facilitator(s)	158	48.8%
	No touch	166	51.2%
Participant’s diagnosis of mental health disorder at the time of the session	Any diagnosis	58	17.9%
	Depression	39	12.0%
	Post-traumatic-stress disorder	16	4.9%
	Anxiety disorder	14	4.3%
	Neurodevelopmental disorder	11	3.4%
	Somatoform Disorder	7	2.2%
	Bipolar Disorder	2	0.6%
	Schizophrenia/Psychotic Disorder	1	0.3%
	Other	23	7.0%

### Descriptive statistics

**Figure 1:**
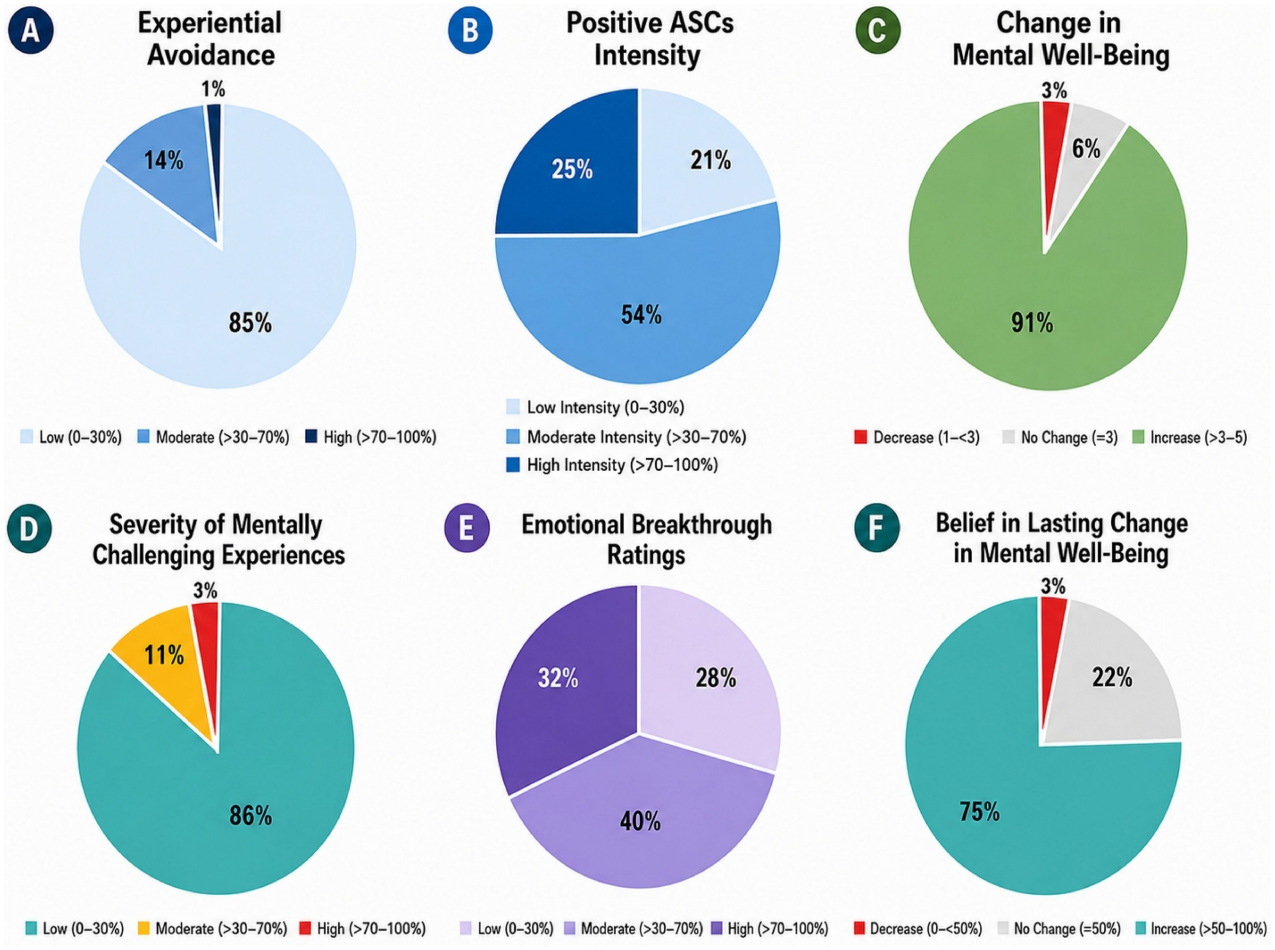
These pie charts show the proportion of participants reporting low, moderate, or high levels on each scale of interest. **(A)** Experiential avoidance ratings; **(B)** positive altered states of consciousness (ASCs) intensity; **(C)** change in mental well-being; **(D)** severity of mentally challenging experiences; **(E)** emotional breakthrough ratings; **(F)** belief in lasting change in mental well-being. Ratings were indicated as low (0–30%), moderate (>30–70%), or high (>70–100%) for all scales except for mental well-being. Warwick–Edinburgh Mental Well-Being Scale change scores were categorized as a decrease (1–<3), no change (=3), or an increase (>3–5), whereas perceived lasting change in mental well-being was categorized as a decrease (0–<50%), no change (=50%), or an increase (>50–100%).

Descriptive statistics for the WEMWBS, 11D-ASCs, EBI, APEQ-S, and the self-designed CES are presented in Table 3. Participant distributions across rating levels on key psychological measures are presented in Figure 1. Participants generally reported low levels of EA during the session, with 76.5% scoring between 0 and 20%. Moreover, CES-Severity was considerably low, with 32.1% of participants indicating no severity (0%), and 54% low severity (>2.5–30%). Different qualities of the session, including perceived support, safety, guidance, and overall experience, are presented in Table 4.

**Table 3:** Descriptive statistics for scales of interest.

Scale	*n*	Minimum	Maximum	*M*	*SE*	*SD*
Experiential avoidance	324	0	84.5	13.94	0.93	16.70
Avoidant responses	324	0	100	19.47	1.2	21.56
Pro-avoidance insights	324	0	91.33	9.39	0.89	15.94
WEMWBS	323	2.2	5	3.84	0.03	0.55
11D-ASCps	323	0	100	48.18	1.56	28.00
Experience of unity	324	0	100	44.69	1.69	30.36
Spiritual experience	324	0	100	45.01	1.73	31.09
Blissful state	324	0	100	56.93	1.75	31.46
Insightfulness	323	0	100	48.37	1.72	30.95
CES	324	0	82.5	13.80	1.02	18.37
EBI	322	0	100	50.35	1.69	30.36

Experiential Avoidance is assessed by the subscales avoidant responses and pro-avoidance insights of the Acceptance/Avoidance-Promoting Experiences Questionnaire (APEQ-S). WEMWBS = Warwick-Edinburgh Mental Well-Being Scale. 11D-ASCps = positive scales of the 11-dimensional Altered States of Consciousness Rating Scale. CES = self-developed Challenging Experiences Scale. EBI = Emotional Breakthrough Inventory.

**Table 4:** Participant ratings of breathwork session qualities (*N* = 324).

Item (scale ranging from 0–100%)	*M*	*SD*
How would you rate your general mindset going into the breathwork session? (very negative to very positive)	81.7%	20.2%
Prior to the session, there were sufficient feelings of trust and support from the facilitator(s). (completely disagree to completely agree)	86.9%	21.8%
Prior to the session, there were sufficient opportunities to discuss questions and concerns. (completely disagree to completely agree)	80.6%	23.4%
Prior to the session, there was enough relevant information on high ventilation breathwork.* (completely disagree to completely agree)	81.7%	24.3%
During the breathwork session, there was sufficient emotional and physical safety and support as needed. (completely disagree to completely agree)	89.5%	18.9%
After the session there were sufficient possibilities to integrate and share your experiences with others. (completely disagree to completely agree)	77.8%	29.0%
After the session there was sufficient guidance regarding physical and mental aftercare after the session. (completely disagree to completely agree)	64.3%	34.6%
Overall, was the experience rather pleasant or rather unpleasant for you? (very unpleasant to very pleasant)	75.6%	23.1%
Would you describe this breathwork session as overall beneficial or harmful for you? (very harmful to very beneficial)	85.4%	18.6%
Do you believe that this breathwork session has led to a lasting change in your sense of well-being? (decreased very much to increased very much)	83.3%	22.3%

*E.g., information on how to breathe during the session, possible physical and mental effects of breathwork, potential benefits and difficulties during the practice, guidance on post-session integration.

### Exploratory analyses of experiential avoidance

Bootstrapped independent t-tests revealed no significant gender differences in EA, *t*(309) = 0.87, *p_boot_* = 0.349. Moreover, EA was not related to the number of prior breathwork sessions, *r* = −0.062, *p_boot_* = 0.134. However, higher age correlated with lower EA, *r* = −0.223, *p_boot_* < 0.001; and participants with a current mental health diagnosis reported higher EA than those without, *t*(322) = −2.08, *p_boot_* = 0.019. Higher EA was also associated with lower perceived facilitator trust and support (*r* = −0.169, *p_boot_* < 0.001), less information on potential physical effects (*r* = −0.272, *p_boot_* < 0.001), reduced emotional safety (*r* = −0.344, *p_boot_* < 0.001), and lower available support during the session (*r* = −0.255, *p_boot_* < 0.001).

### Altered states of consciousness predict experiential avoidance

**Figure 2:**
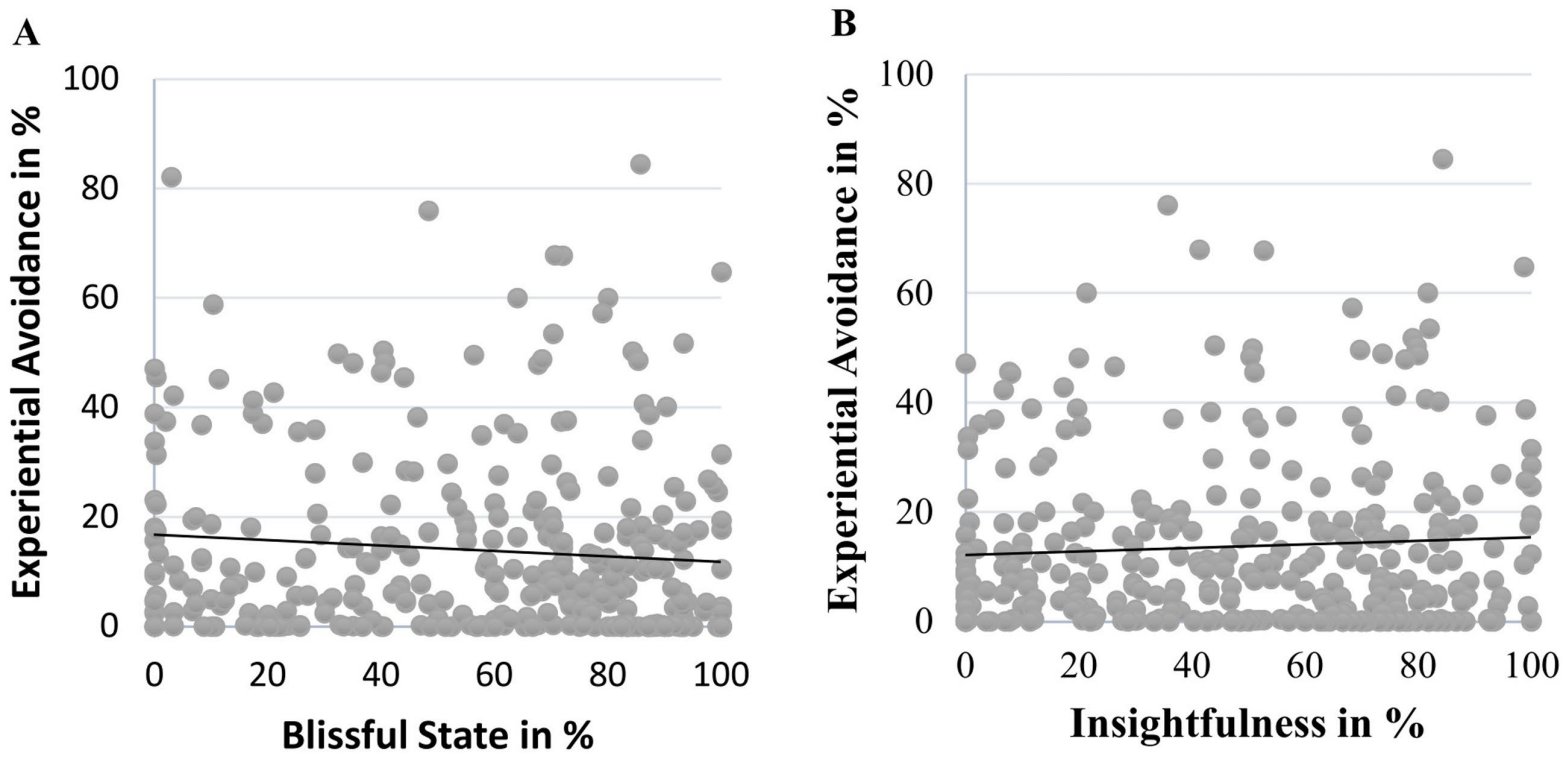
Prediction of experiential avoidance by blissful state **(A)** and insightfulness **(B)**. Higher values represent stronger characteristics in the constructs. Each dot represents an individual participant. Solid lines represent statistically significant predictions (*p* < 0.05).

To test whether ASCs predicted EA during the session (*H2*), a multiple linear regression with the positive 11D-ASC subscales *experience of unity*, *spiritual experience*, *blissful state*, and *insightfulness* as predictors were conducted. One participant was excluded due to missing 11D-ASC data, and 11 participants were excluded due to standardised residuals exceeding ±3 *SD*. Studentised residuals indicated heteroscedasticity and non-normality (Shapiro–Wilk *p* < 0.001). The model significantly predicted EA (*p_boot_* = 0.007), explaining a small proportion of variance (*R^2^* = 0.045). Consistent with the findings above, higher positively valenced experiences were associated with lower EA, with *blissful state* emerging as a significant negative predictor. In contrast, *insightfulness* unexpectedly predicted higher EA, whereas *experience of unity* and *spiritual experience* were non-significant predictors. Figure 2 illustrates the significant associations, with full statistical details for *H2* provided in Table 5. To test the robustness of the findings for *H2*, two separate sensitivity analyses were conducted. In the model adjusted for memory clarity and time since the session, blissful state remained a significant negative predictor of EA (*β* = −0.28, *p* = 0.007). In contrast, insightfulness remained a significant positive predictor (*β* = 0.19, *p* = 0.032). In the model adjusted for BW type, blissful state again remained a significant negative predictor of EA (*β* = −0.33, *p* < 0.001), whereas insightfulness was no longer statistically significant (*β* = 0.15, *p* = 0.098). These findings suggest that the association between blissful state and EA was robust across both sensitivity analyses, whereas the association between insightfulness and EA was less robust when adjusting for BW type.

**Table 5:** Regression estimates for positive 11D-ASC subscales as predictors of experiential avoidance (*H2*).

Model	*F*	*df*1	*df*2	*p_boot_*	*R^2^*	Adjusted *R^2^*
Overall model	3.60	4	307	0.007**	0.045	0.032

Criterion	Predictor	*B*	*SE*	*P_boot_*	95% *CI_boot_*
Experiential avoidance	Experience of unity	0.02	0.05	0.659	[−0.07, 0.13]
	Blissful state	−0.15	0.04	<0.001***	[−0.23, −0.07]
	Insightfulness	0.08	0.03	0.015*	[0.02, 0.15]
	Spiritual experience	0.02	0.04	0.667	[−0.06, 0.09]

11D-ASC = 11-dimensional Altered States of Consciousness Rating Scale. Significance levels: *p < 0.05; **p < 0.01; ***p < 0.001.

### Experiential avoidance predicts lower mental well-being but not emotional breakthrough

**Figure 3:**
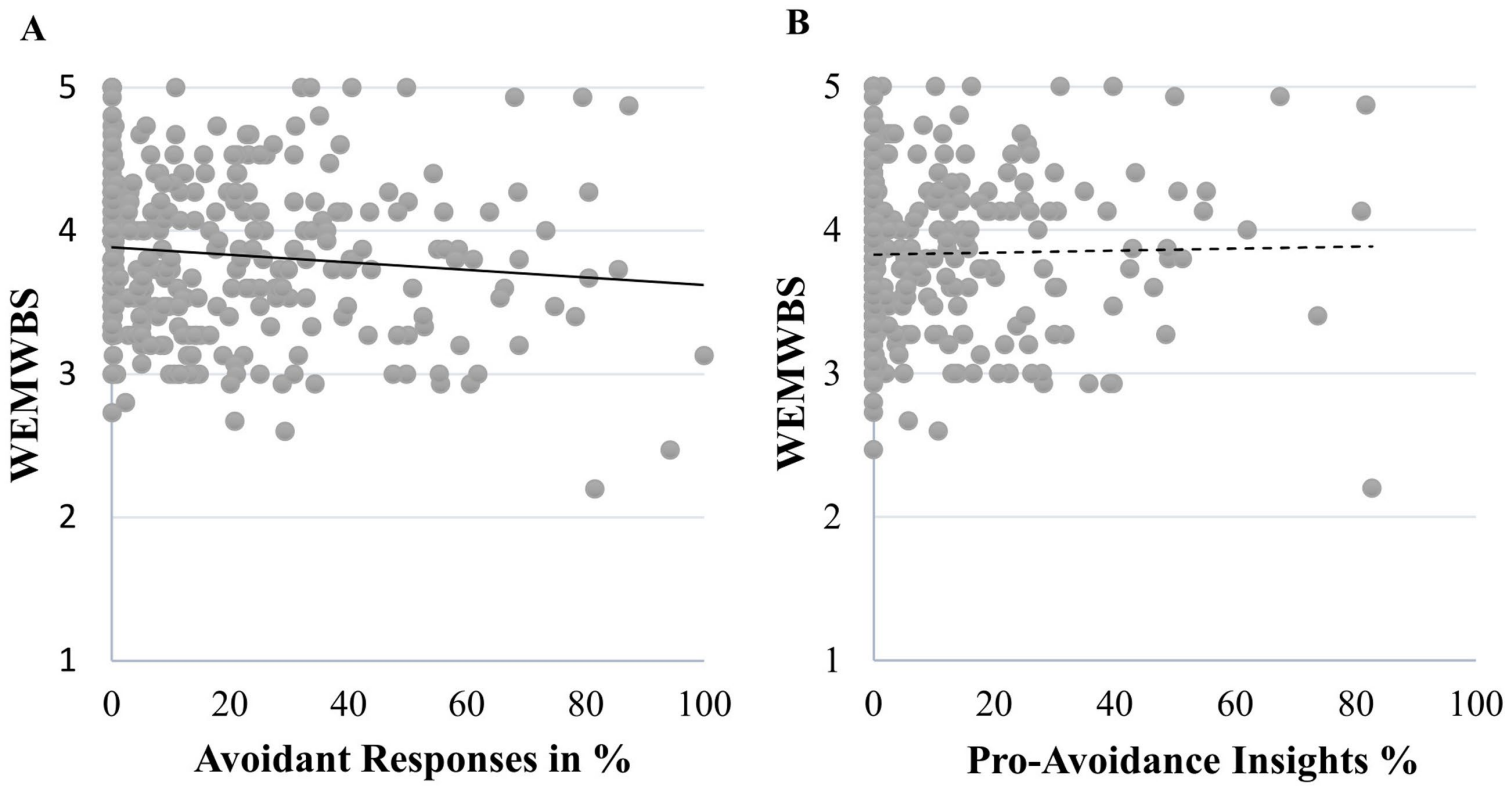
Prediction of change in mental well-being by avoidant responses **(A)** and pro-avoidance insights **(B)**. Each dot represents an individual participant. Solid lines represent significant predictions (*p* < 0.05), whereas dashed lines represent non-significant predictions (*p* > 0.05). WEMWBS = Warwick-Edinburgh Mental Well-Being Scale.

To test *H1* and *H5*, separate multiple linear regressions were conducted with the APEQ-S subscales *avoidant responses* and *pro-avoidance insights* as predictors. For these analyses, full statistical details are provided in Table 6. For *H1*, one participant was excluded due to missing WEMWBS data. The overall model significantly predicted mental well-being, *F* (2, 320) = 3.63, *p_boot_* = 0.028, although it explained only a small proportion of variance (*R^2^* = 0.022; adjusted *R^2^* = 0.016). *Avoidant responses* significantly predicted lower mental well-being, whereas *pro-avoidance insights* were a non-significant predictor. Figure 3 illustrates these relationships. Sensitivity analyses for *H1* indicated that these findings were robust. In the model adjusted for memory clarity and time since the session, avoidant responses remained a significant negative predictor of mental well-being (*β* = −0.18, *p* = 0.008). In contrast, pro-avoidance insights remained a significant positive predictor (*β* = 0.21, *p* = 0.003). In the model adjusted for BW type, avoidant responses again remained a significant negative predictor of mental well-being (*β* = −0.19, *p* = 0.007), whereas pro-avoidance insights remained a significant positive predictor (*β* = 0.19, *p* = 0.009).

**Table 6:** Regression estimates for pro-avoidance insights and avoidant responses as predictors of mental well-being and emotional Breakthrough (*H1* & *H5*).

Criterion	Predictor	*B*	*SE*	*p_boot_*	95% *CI_boot_*
WEMWBS	Pro-avoidance insights	0.005	0.003	0.093	[−0.001, 0.010]
	Avoidant responses	−0.005	0.002	0.007**	[−0.008, −0.001]
Belief in lasting change in mental well-being	Pro-avoidance insights	0.09	0.11	0.370	[−0.112, 0.294]
	Avoidant responses	−0.26	0.08	<0.001***	[−0.404, −0.098]
EBI	Pro-avoidance insights	0.220	0.140	0.105	[−0.039, 0.489]
	Avoidant responses	−0.175	0.100	0.069	[−0.353, 0.016]

WEMWBS = Warwick-Edinburgh Mental Well-Being Scale. EBI = Emotional Breakthrough Inventory. Significance levels: *p < 0.05; **p < 0.01; ***p < 0.001.

**Figure 4:**
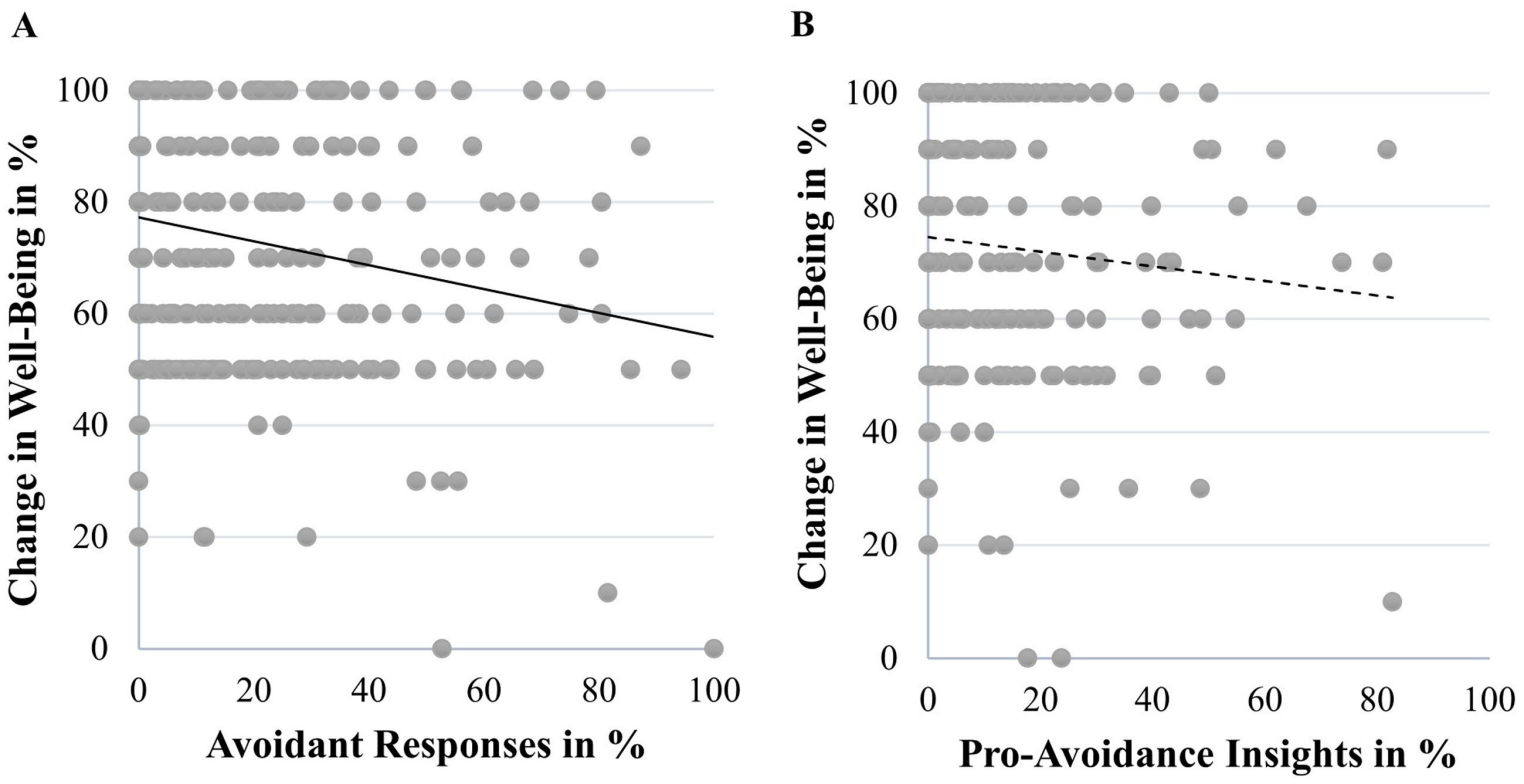
The prediction of belief in lasting change in mental well-being due to the breathwork session by avoidant responses **(A)** and pro-avoidance insights **(B)** is depicted. The belief in a lasting improvement in mental well-being was measured by the single item “Do you believe that this breathwork session has led to a lasting change in your sense of well-being” from 0% (decreased very much) to 100% (increased very much). Each dot represents an individual participant. Solid lines represent significant predictions within the multiple linear regression (*p* < 0.05), whereas dashed lines represent non-significant predictions (*p* > 0.05).

To further examine how the same EA subscales relate to participants’ evaluations of the session, we conducted an exploratory regression to assess whether EA predicted the belief that the session produced lasting improvements in participants’ mental well-being. Studentised residuals were non-normally distributed (Shapiro–Wilk *p* < 0.001). The model significantly predicted belief in lasting change, *F* (2, 321) = 7.65, *p_boot_* < 0.001, again explaining a small proportion of variance (*R^2^* = 0.045; adjusted *R^2^* = 0.032). Consistent with the previous analysis, the *avoidant responses* subscale was a significant negative predictor, whereas the *pro-avoidance insights* subscale was a non-significant predictor. These associations are shown in Figure 4.

**Figure 5:**
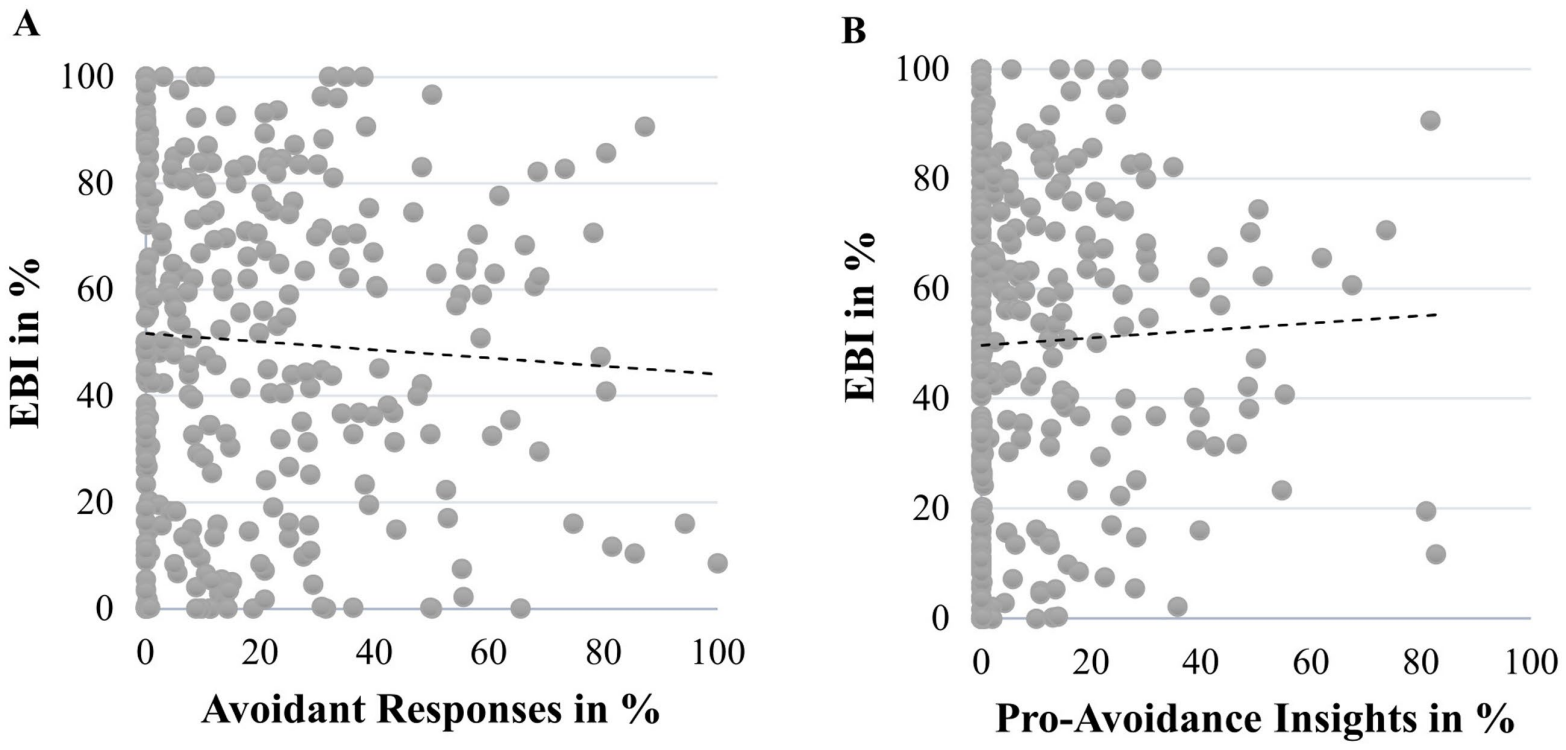
Non-significant prediction of emotional breakthrough via avoidant responses **(A)** and pro-avoidance insights **(B)**. Each dot represents an individual participant. Dashed lines represent non-significant predictions (*p* > 0.05). EBI = Emotional Breakthrough Inventory.

For *H5*, *n* = 2 participants were excluded due to missing EBI data. Studentised residuals were again non-normally distributed (Shapiro–Wilk *p* < 0.001). In contrast to the previous outcomes, the model did not significantly predict emotional breakthrough, *F* (2, 319) = 1.70, *p_boot_* = 0.184, accounting for minimal variance (*R^2^* = 0.011; adjusted *R^2^* = 0.004). Neither *avoidant responses* nor *pro-avoidance insights* significantly predicted emotional breakthrough experiences. Figure 5 visualizes these associations.

### Experiential avoidance does not mediate the relationships between acute experiences and mental well-being

**Figure 6:**
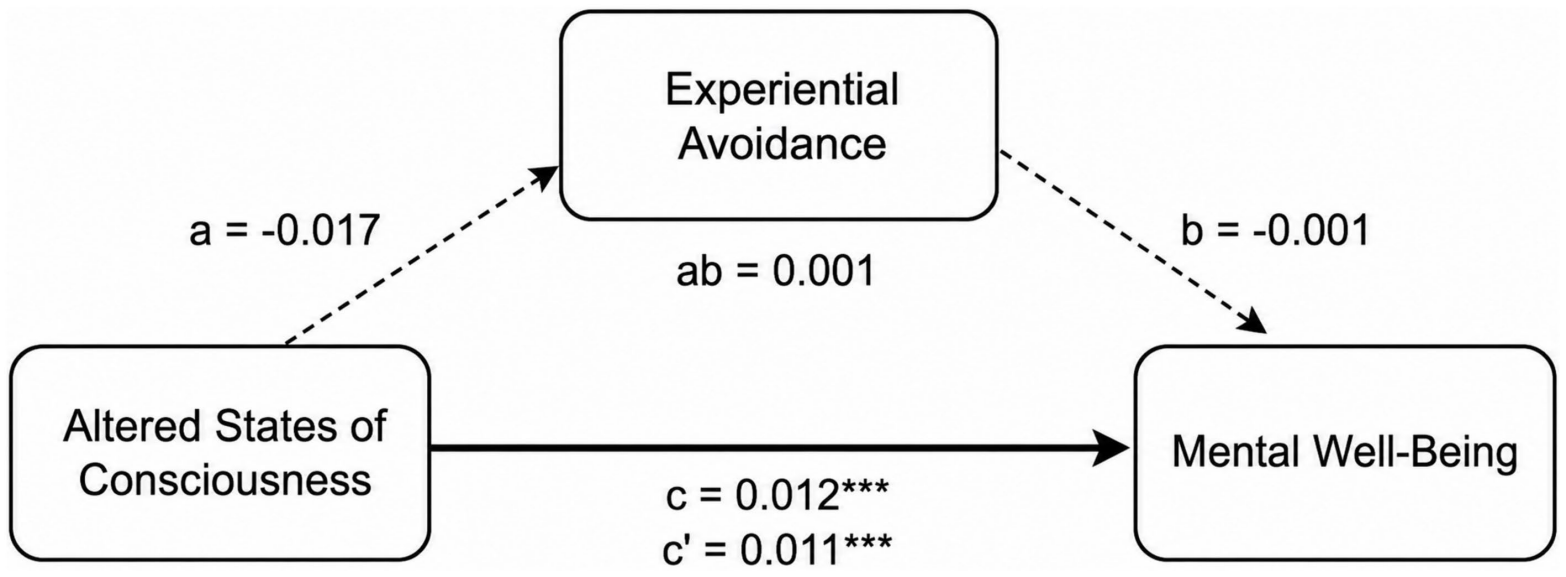
Non-significant mediation between altered states of consciousness and mental well-being with Experiential Avoidance as a mediator. Unstandardised effects of a simple mediation analysis are depicted to illustrate the relationship between the independent variable altered states of consciousness (ASCs), the mediator variable experiential avoidance (EA) and the dependent variable mental well-being; a = effect of ASCs on EA; b = effect of EA on mental well-being; c = total effect and c’ = direct effect of ASCs on mental well-being; *p < 0.05; **p < 0.01; ***p < 0.001. Solid arrows represent significant effects (*p* < 0.05) and dashed lines represent non-significant effects (*p* > 0.05).

Simple mediation analyses with heteroscedasticity-consistent inference (Davidson and MacKinnon, 1993) were conducted to test *H3* and *H4* (full mediation statistics are reported in Tables 7, 8). For *H3*, 13 participants were excluded due to missing data (*n* = 2) or residual outliers (*n* = 11; > 3 *SD*). Residuals showed heteroscedasticity and non-normality (Shapiro–Wilk, all *p* < 0.001). ASCs significantly predicted higher post-session mental well-being. In contrast, ASCs did not predict EA, and EA did not predict mental well-being. The indirect effect was non-significant. This suggests that the link between breathwork-induced ASCs and mental well-being was not significantly mediated by experiential avoidance (Figure 6).

**Table 7:** Statistical parameters for the mediation model linking altered states of consciousness, experiential avoidance, and mental well-being (*H3*).

Path	*B*	*SE*	*p_boot_*	95% *CI_boot_*
Total effect (c) for 11D-ASCps → WEMWBS	0.012	0.001	<0.001***	—
11D-ASCps → EA (a)	−0.017	0.028	0.216	—
EA → WEMWBS (b)	−0.001	0.002	0.229	—
Indirect effect (a × b)	0.001	0.001	—	[−0.001, 0.003]
Direct Effect (c‘)	0.011	0.001	<0.001***	—

11D-ASCps = positive subscales of the 11-dimensional Altered States of Consciousness Rating Scale. WEMWBS = Warwick-Edinburgh Mental Well-Being Scale. EA = Experiential Avoidance. Significance levels: *p < 0.05; **p < 0.01; ***p < 0.001.

**Table 8:** Statistical parameters for the mediation model linking mentally challenging experiences, experiential avoidance, and mental well-being (H4).

Path	*B*	*SE*	*p_boot_*	95% *CI_boot_*
Total effect (c) for CES → WEMWBS	−0.004	0.002	0.035*	—
CES → EA (a)	0.275	0.072	<0.001***	—
EA → WEMWBS (b)	−0.002	0.002	0.444	—
Indirect effect (a × b)	−0.001	0.001	—	[−0.002, 0.001]
Direct Effect (c‘)	−0.003	0.002	0.076	—

CES = self-developed challenging experiences scale. WEMWBS = Warwick-Edinburgh Mental Well-Being Scale. EA = Experiential Avoidance. Significance levels: *p < 0.05; **p < 0.01; ***p < 0.001.

**Figure 7:**
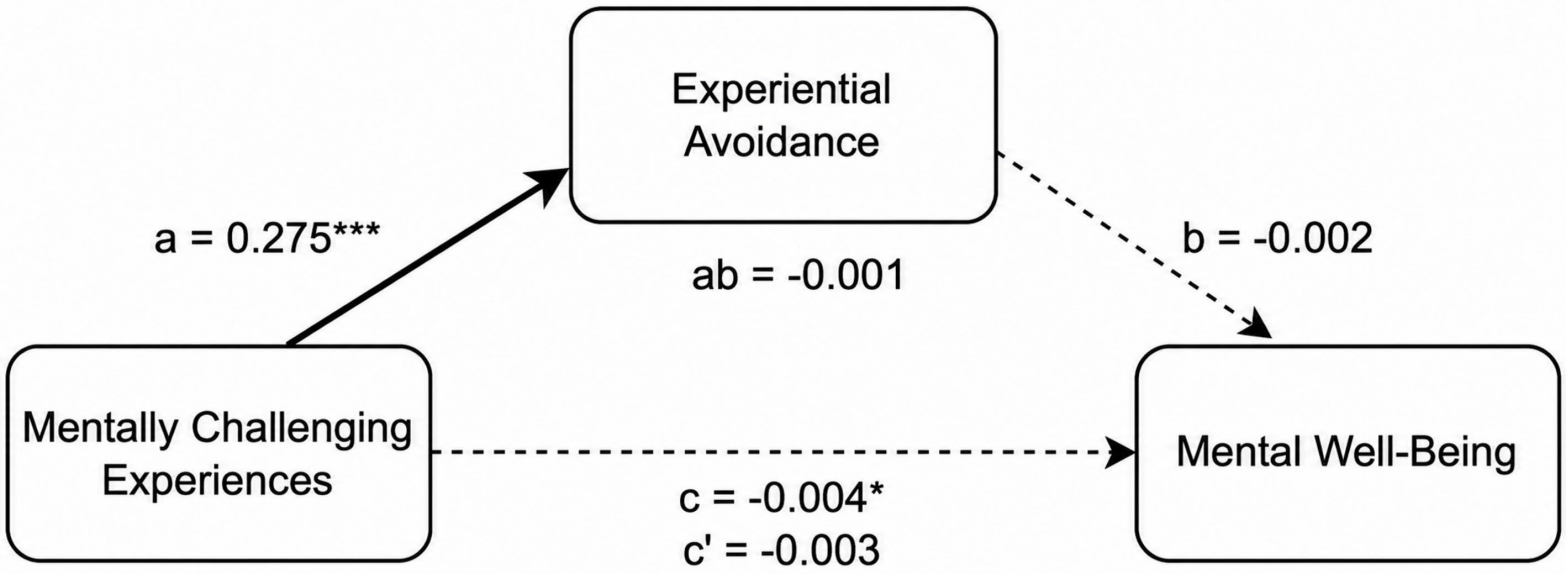
Non-significant mediation between challenging experiences and mental well-being with Experiential Avoidance (EA) as a mediator. Unstandardised effects are depicted to illustrate the relationship between the independent variable mentally challenging experiences, the mediator variable EA and the dependent variable mental well-being; a = effect of challenging experiences on EA; b = effect of EA on mental well-being; c = total effect and c’ = direct effect of challenging experiences on mental well-being; *p < 0.05; **p < 0.01; ***p < 0.001. Solid arrows represent significant effects (*p* < 0.05) and dashed lines represent non-significant effects (*p* > 0.05).

For *H4*, eight participants were excluded due to missing data (*n* = 1) or outliers (*n* = 7). Residuals again showed heteroscedasticity and non-normality (Shapiro–Wilk *ps* < 0.05). Challenging experiences significantly predicted lower mental well-being. Moreover, mentally challenging experiences significantly predicted higher EA. However, EA did not significantly predict mental well-being, and the indirect effect was non-significant, indicating that EA did not mediate the relationship between mentally challenging experiences and mental well-being (Figure 7).

Separate sensitivity analyses for the main findings showed that the overall pattern of results remained robust after adjusting for memory clarity and time since the session as well as after separately adjusting for breathwork type. Specifically, mental well-being remained significantly predicted by ASCs after adjustment for memory clarity and time since the session (*β* = 0.62, *p* < 0.001), and after adjustment for breathwork type (*β* = 0.64, *p* < 0.001). Similarly, mentally challenging experiences remained significantly associated with mental well-being after adjustment for memory clarity and time since the session (*β* = −0.13, *p* = 0.018), and after adjustment for breathwork type (*β* = −0.13, *p* = 0.021).

## Discussion

This cross-sectional international online study found that lower levels of EA during HVB predicted higher mental well-being in the days after the session. Exploratory analyses further confirmed this finding: participants who reported fewer avoidant responses were also more likely to believe that the session had a lasting positive impact on their mental well-being. In both analyses, only the subscale avoidant responses, but not pro-avoidance insights, significantly contributed to these outcomes. Within ASCs, blissful states were associated with reduced EA, whereas higher insightfulness unexpectedly predicted higher EA. However, neither experience of unity nor spiritual experience significantly influenced EA. Mediation analyses further showed that EA did not mediate the relationships between ASCs or mentally challenging experiences and post-session mental well-being. Instead, ASCs exerted a direct positive effect on mental well-being, whereas challenging experiences had a direct negative effect, independent of EA. EA was also unable to significantly predict whether participants experienced an emotional breakthrough during HVB. Together, these findings suggest that although reducing avoidant responses during HVB may support greater mental well-being, EA does not account for the influences of ASCs or mentally challenging experiences, nor does it shape the occurrence of emotional breakthrough experiences. Rather, blissful ASC states may play a central role in reducing avoidance during HVB.

About 80% of participants in the present study reported moderate to high intensities of ASCs, suggesting that HVB can induce ASCs comparable to those observed in psychedelic-assisted therapy, consistent with Eyerman (2013), Uthaug et al. (2019), and Havenith et al. (2025), even though some studies report lower intensities (e.g., Uthaug et al., 2022; Stocker et al., 2023). In the present study, higher ASC intensity, particularly blissful states, predicted lower EA levels during the session. Therefore, it is possible that blissful ASC states, such as feelings of pleasure, love, and inner peace, function as a shaping mechanism, fostering reduced EA and greater acceptance. Conversely, insightfulness unexpectedly predicted higher levels of EA. This pattern could reflect that higher EA sometimes manifests as a cognitive or analytical engagement with the session, in which participants gain intellectual insights without fully engaging emotionally. However, this may also be attributable to a potential overlap between the 11D-ASCs subscale insightfulness and the APEQ-S subscale pro-avoidance insight, as both may encompass processes of reflection, understanding, and learning, along with an inward-looking perspective.

Interestingly, exploratory analyses of the present study suggest that other factors may additionally contribute to reductions in EA. Lower levels of EA were associated with older age, higher perceived trust, support, and emotional safety provided by the facilitator(s), and greater information about the potential physical effects of HVB. These findings highlight the importance of a positive relationship and sense of trust with the facilitator(s) in reducing EA, likely by fostering emotional safety and openness to the experience (Levin et al., 2024). In addition, limited information about potential physical effects (e.g., tingling, changes in heart rate, or muscle cramping; Fincham et al., 2023a) may render these sensations uncertain or threatening, thereby further increasing EA. Nevertheless, the directionality of these relationships remains unclear: higher EA may also lead participants to underestimate support and safety.

Participants in the present study generally rated their most recent HVB session as having a positive impact on their mental well-being, despite variation in the time elapsed since the session, which could be up to 5 years. This finding is consistent with prior research reporting perceived benefits of HVB-related practices (Uthaug et al., 2022; Puente, 2014b; Havenith et al., 2025). Furthermore, the present study provides initial evidence that lower levels of EA during the session are associated with greater retrospectively perceived post-session well-being in the context of HVB. Importantly, participants with a previous mental health diagnosis reported higher levels of EA during their session compared to those without such a history, which may reflect the detrimental role of EA in psychological functioning. Moreover, our findings align with those of Zeifman et al. (2023), who showed that reductions in EA mediated improvements in depression outcomes in the psilocybin but not the escitalopram treatment group.

Although the present study provides initial evidence that lower levels of perceived EA are associated with greater post-session well-being in the context of HVB, the explained variance was relatively modest. One possible explanation for the small effect size is that EA may operate largely outside conscious awareness (Augusto, 2010), limiting the sensitivity of self-report measures to capture actual avoidance. Objective measures, such as the approach–avoidance paradigm, may therefore offer a more accurate assessment of avoidance behaviour (Ikeda, 2022). EA also tends to become clinically relevant only when it is excessive and substantially restricts an individual’s behavioural repertoire (Kashdan et al., 2006; Arnaudova et al., 2017). If EA during an HVB session does not produce enduring negative consequences, it may have little observable effect on subsequent mental well-being (Hofmann and Hay, 2018). Additionally, EA may be more easily enacted in HVB than under psychedelic substances, because participants can regulate or normalize their breathing, thereby rapidly reducing ASC intensity (as seen in Havenith et al., 2025) and preventing the escalation of challenging experiences.

Notably, our findings indicate that EA does not mediate the relationship between ASCs and mental well-being, contrasting with psychedelic research in which increases in psychological flexibility or reductions in EA predict improvements in mental well-being, depression, and anxiety (e.g., Davis et al., 2020; Zeifman et al., 2020). Instead, HVB may enhance mental well-being through direct experiential effects, such as blissful states or insightfulness, rather than through reduced EA (Uthaug et al., 2022; Griffiths et al., 2011). This difference may also reflect sample characteristics, as prior work typically involved clinical populations, whereas our predominantly healthy sample exhibited considerably low levels of EA and mentally challenging experiences. Mentally challenging experiences likewise directly predicted poorer well-being and higher EA, suggesting that individuals encountering more distress during HVB may benefit less from it, consistent with Roseman et al. (2018) and Haijen et al. (2018). However, because EA was assessed retrospectively as an overall session-level experience, it is also possible that avoidance occurred transiently but resolved during the course of the session, for example, as participants shifted toward greater acceptance or emotional engagement. Nevertheless, the absence of EA mediation in both positive and challenging ASC pathways suggests that other mechanisms, such as acceptance or emotional breakthroughs, may better account for HVB’s influence on mental well-being.

The present findings suggest that emotional breakthrough experiences and EA are largely independent constructs. Although Wolff et al. (2022) found significant correlations between the EBI and EA subscales, effect sizes were small in a large sample (*N* = 1,829). Moreover, Zeifman et al. (2023) did not find a significant association between reductions in EA and emotional breakthrough in psychedelic-assisted therapy. Although further research is needed, these findings, consistent with Zeifman et al. (2023), indicate that low EA may not be a prerequisite for emotional breakthrough in HVB. Although it may seem intuitive that lower EA would facilitate breakthrough, as avoidance could prevent engagement with emotions, the relationship may be more dynamic: EA early in a session may not preclude an emotional breakthrough later, particularly if participants subsequently shift towards acceptance. Indeed, Wolff et al. (2022) reported strong links between acceptance and EBI, highlighting that it may be the emergence of acceptance, rather than the absolute absence of avoidance, that enables transformative experiences. Together, these results suggest that temporal fluctuations in EA and the facilitation of acceptance may be more critical than baseline avoidance for understanding how emotional breakthroughs unfold during HVB.

This study has several limitations that should be considered when interpreting the results. First, the study was not conducted under experimental conditions with standardised HVB instructions, researcher-observed sessions, or pre-post assessments of mental well-being. Instead, it relied on participants’ retrospective reports of naturally occurring breathwork sessions that may have varied substantially in technique, setting, and implementation. As these sessions were neither conducted nor observed by the researchers, it was not possible to verify what occurred during them, including whether the accelerated breathing phase lasted at least 15 min. Accordingly, the findings should not be interpreted as evidence of causal effects of HVB on mental well-being, particularly given the cross-sectional design, retrospective self-report methodology, potential self-selection biases, and assessment of all key constructs within the same survey session, which may introduce shared method variance. Any conclusions regarding the potential effects of HVB should therefore remain cautious and provisional. Moreover, participants drew from various breathwork schools, adding heterogeneity in techniques and contexts. Consequently, the present study does not allow conclusions about the effects of one specific breathwork technique. However, sensitivity analyses adjusting for breathwork type did not materially change the relevant findings. Retrospective self-reports may have been affected by memory bias, mood, or personality, and 11% recalled the session only “somewhat clearly,” reducing internal validity. In addition, participants were asked to report HVB experiences that may have occurred up to five years before study participation. Although sensitivity analyses adjusting for memory clarity and time since the session did not materially alter the results, recall bias cannot be excluded. Regarding external validity, recruitment reached 23 countries in a naturalistic setting. However, the sample was predominantly educated, German-speaking, and non-clinical, limiting generalisability. Low average scores in EA and possible ceiling effects in mental well-being may have constrained outcome variability. Only two APEQ-S subscales were used, omitting the acceptance-related experience subscale, and the measure itself requires further validation. Although mediation models were applied, the cross-sectional design limits causal interpretations, particularly regarding the assumed temporal sequence of psychological states during breathwork. The complexity of breathwork sessions complicates measurement, as participants might experience avoidance, blissful states, challenging experiences, and emotional breakthroughs at different times within the same session.

Taken together, these limitations highlight the need for future research to replicate and extend our findings on the potential mental health benefits of reduced EA and enhanced ASCs during HVB. Future studies should use prospective, lab-based, or randomised designs with standardised HVB protocols, appropriate control conditions, and pre-post measurements of mental well-being to clarify potential mechanisms and causal effects. Such work should employ longitudinal designs and objective measures of avoidance to clarify how HVB influences EA, psychological flexibility, and mental well-being over time. Comparative studies with other therapeutic modalities may further determine whether HVB confers unique advantages, particularly for clinical populations. Additionally, future research should examine how EA relates to mental health conditions such as anxiety and depression and incorporate broader emotion-regulation strategies, including acceptance.

## Conclusion

In conclusion, the current findings suggest that EA plays a small but significant role in the context of HVB. Participants’ tendencies to avoid challenging emotions, thoughts, or memories during breathwork may explain why some individuals benefit more or less from practices such as Holotropic or Conscious Connected Breathwork. Due to the importance of overcoming EA in a variety of mental disorders, HVB might mirror mechanisms of cognitive behavioural therapy, as proposed for psychedelic-assisted therapy (Wolff et al., 2020). Breathwork facilitators should continue encouraging participants to “let go” during HVB sessions to help them move from a defensive to an accepting posture. Moreover, breathwork facilitators should ensure an atmosphere of trust, support, and emotional safety, and provide information about the physical effects of HVB to reduce EA. Due to its ability to improve mental well-being and its similarities to pharmacologically induced ASCs, HVB might provide a low-threshold alternative to psychedelic-assisted therapy in the future.

## Data Availability

The raw data supporting the conclusions of this article will be made available by the authors, without undue reservation.
